# Ethnomedicinal use of African pangolins by traditional medical practitioners in Sierra Leone

**DOI:** 10.1186/1746-4269-10-76

**Published:** 2014-11-20

**Authors:** Maxwell K Boakye, Darren W Pietersen, Antoinette Kotzé, Desiré L Dalton, Raymond Jansen

**Affiliations:** Department of Environmental, Water and Earth Sciences, Tshwane University of Technology, P/Bag X680, Pretoria, 0001 South Africa; African Pangolin Working Group (APWG), P/Bag X680, Pretoria, South Africa; National Zoological Gardens of South Africa, P.O. Box 754, Pretoria, 0001 South Africa; Department of Genetics, University of the Free State, P.O. Box 339, Bloemfontein, 9300 South Africa

**Keywords:** Pangolin scales, Pangolin body parts, Spiritual ailments, Conservation, Sierre Leone

## Abstract

**Background:**

Pangolins (Manidae) have long been used for traditional medicinal purposes in most parts of sub-Saharan Africa. However, very little is known about the extent of this use, the body parts that are used and the ailments these practices are attempting to cure or alleviate. Pangolin body parts are used extensively and frequently by traditional medical practitioners in Sierra Leone.

**Methods:**

A total of 63 traditional medical practitioners consented and were interviewed using semi-structured questionnaires on the traditional medicinal use of pangolin body parts. The use value, informant agreement ratio and use agreement value for each pangolin part was calculated to ascertain the most sought after body part, the level of knowledge dissemination among traditional medical practitioners about body parts and the most culturally significant body part.

**Results:**

It was found that 22 pangolin parts are used to treat various ailments and conditions under 17 international categories of diseases. The highest use value was recorded for scales while eyes had the highest level of consensus among the traditional medical practitioners. The highest use value and informant agreement ratio for scales were recorded for spiritual ailments. Scales were the most culturally significant body part according to the use agreement value.

**Conclusion:**

This study indicates a high importance value for pangolins as part of these communities’ spiritual, cultural and medicinal beliefs. However, the numbers of individuals harvested from the wild remains unknown and unregulated even though pangolins have been listed under Schedule 2 of the Wildlife Conservation Act, 1972, of Sierra Leone, which prohibits any person from hunting or being in possession of pangolins. It is likely that this unregulated harvesting and poaching of this threatened species, for medicinal purposes, is unsustainable and there is an urgent need to determine pangolin population abundance within this region to ensure their sustainable harvesting for cultural use and conservation.

## Background

Traditional medicine dominates medical systems available to millions of individuals and remains the main, if not the only, source of medical care for the majority of people in the developing world [1–3]. Animal-based remedies constitute an integral part of African traditional medicine both in rural and urban areas. An estimated 80% of the continent’s population depend on traditional medicine for their healthcare needs [2–4]. The people of Sierra Leone are no exception to this. The majority of the population of Sierra Leone depend on traditional medicine to meet their healthcare needs [5]. Some 90% of deliveries at community level are attended by traditional birth attendants who are an integral component of traditional medical practice [5]. Shackman and Price [6] also found that 90% of patients with mental health problems have received treatment from traditional healers in the Northern Province of Sierra Leone. Indeed, traditional medicine is the first level of contact for most Sierra Leoneans due to inadequate healthcare facilities and affordability when these are available.

Traditional medical practitioners are highly dependent on harvesting from the biodiversity, and primary ingredients used by the traditional healers are wild animals, plant species and mineral resources for the preparation of their therapeutic remedies for their patients [1, 7, 8]. Animals have been documented as a source of medicine throughout human history. According to Weiss [9], Anageletti et al. [10], Lev [11] Alves and Alves [12], Alves and Sauto [13], animal-based medicines have been used since time immemorial. Most Africans attach special magical healing power to wild animals and their by-products used in traditional medicine [14–21].

A mammal that has long been used for traditional medicinal purposes throughout Africa is the pangolin [15–17, 19, 22–26] and also traditional medicinal systems in Asia [27–29]. Pangolins or scaly anteaters belong to the order Pholidota [30]. They are covered with keratinous scales and have adapted to a specialised diet of ants and termites [15, 31–33]. They feed by burrowing with their claws adapted for raiding ant and termite mounds, aided by their highly modified elongated and sticky tongue specialised for consuming ants and termites [31–33]. They are usually solitary and inhabit burrows or inside tree holes, and have a very slow reproduction rate [31, 33]. When threatened, they curl their body up like a ball in a defensive posture [15, 33].

All extant species of pangolin (four Asian species and four African species) are listed in Appendix II of CITES. The black-bellied pangolin (*Phataginus tetradactyla*), white-bellied pangolin (*Phataginus tricuspis*) and the giant ground pangolin (*Smutsia gigantea*) are distributed throughout Sierra Leone [34]. They occur mainly in forests and forest mosaics in moist and semi-deciduous forest habitat type. No previous studies have investigated the use of pangolins by traditional medical practitioners in Sierra Leone and this is the first attempt to document the use of whole or parts of pangolins in the treatment of human-related diseases. In addition, this study investigates implications of pangolin use by traditional medical practitioners on the conservation status and potential threat to African pangolin biodiversity.

## Materials and methods

### Study site

**Figure 1:**
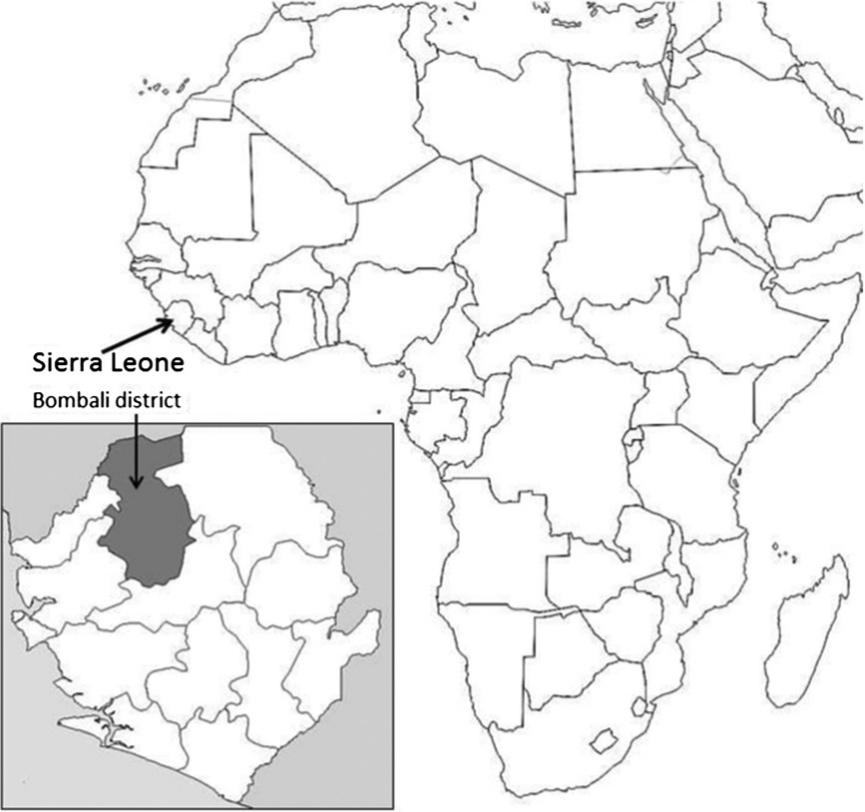
**Map of Africa indicating Sierra Leone and the study district of Bombali.**

The study was conducted in the Bombali district (9°20′N and 12°15′W) of Sierra Leone (Figure 1). This district has a human population of 408,390 [35] and occupies a total area of 7 985 km^2^ [36]. The district shares borders with the Republic of Guinea to the north, Port Loko and Kambia districts to the west, Tonkolili district to the south and Koinadugu district to the east. Ethnically, the Temne and Limba form the largest ethnic groups in Bombali district. Makeni is the headquarters and largest town in Bombali district.

The Bombali district was chosen for this study because it is reported by the Justice and Peace and Human Rights Commission [37] as having the poorest level of health services in Sierra Leone. The Development Assistance Coordinating Office also classified Bombali district as the worst in secondary and primary healthcare services when compared with other districts nationwide and, as a result, the inhabitants of Bombali district depend heavily on traditional medical practitioners for their medical needs [6]. For the purposes of this study TMP is referred to those people who are considered to be healers by the district Traditional Healers Association by virtue of their membership with the association.

### Data collection

Ethnozoological data on medicinal use of pangolins was collected using semi-structured interviews held between the months of October to November 2013. During the first contacts with the local population, a meeting was held with the local tribal leader to explain the research and to obtain informed consent to conduct the study. The local tribal leader then introduced the researcher to the chairman of the local association of traditional healers. The objective of the research was explained to the chairman, who called a meeting of the association members to explain the project to them. The sampling process was purposeful with participants intentionally selected because they could provide the information about the issues identified as important to this study [38]. Of the 100 traditional medical practitioners requested to participate in this study, 98 members confirmed they are or have used whole or parts of pangolins in their remedies. Of these, 63 agreed to participate in this study with informed consent. Participants were made aware of their rights to decide to either voluntarily participate in the study or decline. Semi-structured interviews were conducted to obtain information from respondents. The interview questions focused on the interviewees’ knowledge of the uses for pangolin parts or whole pangolins for medicinal purposes to cure or alleviate a particular medical ailment or set of ailments. Verbal prompts and probes were used to motivate informants and elicit information from them. The pace and direction of the interviews were dictated by the participants. Participants were shown photographs of the three African pangolin species distributed in Sierra Leone. The key questions that traditional medical practitioners were asked were: do you know the animal called pangolin (participants were requested to provide the local name) and do you know whether it serves as medicine? In the semi-structured interviews, the traditional medical practitioners (interviewees) were requested to name particular species of pangolin and their parts used as medicine, the ailments for which that body part was prescribed and how the animals was obtained. Traditional medical practitioners were also questioned on how this knowledge was acquired. All interviews were conducted in Krio English language which is the most widely spoken language in Sierra Leone, with a local Sierra Leonean translator translating if the interviewee did understand certain aspects of the Krio English language.

### Data analysis

Three quantitative value indices: Use values (UV), informant agreement ratio (IAR) and use agreement value (UAV) were calculated.

#### Use value (UV)

The use value (UV) for each pangolin part was calculated employing a formula modified from Albuquerque et al. [39], which uses animal parts instead of the whole individual. This approach is based on a quantitative method to demonstrate the importance of species parts given by a local population [40]. The UV of each pangolin part mentioned was calculated using the formula:


where: Up = the number of uses mentioned by each informant for a given pangolin part, n = the total number of informants [39, 41].

#### Informant agreement ratio (IAR)

The Informant Agreement Ratio (IAR) was used to measure the consensus level among informants for pangolin body part. The original formula proposed by Trotter and Logan [42] was interpreted as follows:


where *nr* is the total number of medicinal responses registered for pangolin body part and *nc* is the number of disease categories that are treated with this body part. The number of disease categories (*nc*) was calculated as the number of the International Classification of Diseases (ICD) categories (Version 10) [43] that informants claimed to treat. Sixteen ICD categories were identified but spiritual ailments were added because it plays an important role as causes of diseases in African folk health systems. The total number of ICD categories in this study was 17. The IAR of a medicinal part varies between 0 (when the number of health conditions treated equals the number of medicinal responses) and 1 (whereby all participants agree upon the exclusive use of the part for a particular health condition).

#### Use agreement value (UAV)

According to Thomas et al. [44] this index provides a valid and easily derived estimation of a medicinal resources cultural significance. Following the suggestions of [44], but using UV instead of Quality Use Value (QUV), the use agreement value (UAV) was defined as follows:


where UV is the use value of body part and IAR is the informant agreement ratio of a particular body part.

## Results

Regarding gender of participants, 90% (n = 57) were males and 10% (n = 6) were females. With regard to the age of participants, 37% (n = 23) were above 60 years, and 2% (n = 1), 14% (n = 9) and 48% (n = 30) were between 31–40, 41–50 and 51–60 years old, respectively. Regarding the number of years of practice, 68% (n = 43) had practised for more than 20 years, 30% (n = 19) between 16–20 years and 2% (n = 1) between 11–15 years. With regard to formal education, 41% (n = 26) of the interviewees were illiterate, 57% (n = 36) had attended school but their education was below secondary level and 2% (n = 1) had attended school above secondary level. All the interviewees classified themselves as full-time traditional medical practitioners.

A total of 22 pangolin body parts were identified as being used and prescribed for the treatment of 59 diseases and ailments (Table 1). The scales of pangolins had the highest use report (UR), followed by oil meat, head and tail, respectively (Table 1). The throat, sex organs (both male and female) and liver had the lowest UR whereas the eyes, forefoot, thorax, toes and whole animal had the same UR. The scales, head, meat and tail were the body parts with the highest number of ICD category use reports, respectively (Table 1). The scales were used in 12 of the 17 ICD categories, the head and tail in 10 categories and meat in 9 categories.

**Table 1:** **Use of pangolin body parts and the prescribed conditions treated, Use Report (UR) and International Classification of Diseases (ICD) categories**

Parts	Conditions treated	Use report	ICD categories
Scales	Skin disease, impotence, infertility, broken ribs, stomach diseases, inflammation of the navel, bulletproof, cutlassproof, athlete's foot, claw hand, nail disorders, healing premature babies, heel fissure, arthritis, rheumatism, epilepsy, ear infection, body pain, elephantiasis, waist pain, healing wound, skin rash, protection from witchcraft, broken bones, spiritual protection and skin scars	70	12
Oil	Skin rash, skin stretch marks, heel fissure, skin diseases, knee pain, skin scars, heart diseases, claw hand, elephantiasis and body ache	27	5
Meat	Healing premature babies, stomach diseases, rheumatism, epilepsy, high blood pressure, for increasing intelligence, body pain, common childhood diseases, convulsion and anaemia	23	9
Head	Infertility, headache, antidote for poison, skin diseases, mental illness, toothache, heart disease, paralysis, hernia and claw hand	21	10
Tail	Impotence, spiritual protection, Apollo (acute haemorrhagic conjunctivitis), paralysis, claw hand, convulsion, fainting, stomach diseases, protection against snake bite, curse sting from scorpion, waist pain, elephantiasis and heel fissure	20	10
Foot	Heel fissure, back pain, elephantiasis, athlete's foot and broken bones	7	4
Tongue	Rheumatism, stop/control bleeding, mental illness, piles and fainting	7	4
Bones	Skin scars, healing wounds, stiffness of joints, rheumatism and joint pains	7	3
Blood	Healing wounds, elephantiasis, rheumatism, stomach diseases, heart diseases and protection against witchcraft	7	6
Male sex organ	Hernia, headache, elephantiasis, athlete's foot, infertility and impotence	6	5
Intestines	Stomach diseases, headache and for good luck	5	3
Claws	Protection from witchcraft, skin stretch marks, asthma and heart burn	4	4
Brain	Heart diseases, mental illness and stomach diseases	4	3
Heart	Stomach diseases and heart diseases	3	2
Whole animal	Leprosy and invisibility	2	2
Toes	Apollo and epilepsy	2	2
Thorax	Chest pain and broken ribs	2	2
Forefoot	Impotence and elephantiasis	2	2
Eyes	Apollo	2	1
Throat	Goitre	1	1
Sex organ (both male and female)	Infertility	1	1
Liver	Asthma	1	1

Table 2 indicates the contribution of each ICD category to the total use value index (UV) of the 22 pangolin body parts used by the traditional healers. The highest UV of the scales was for spiritual ailments while the highest UV of tissue oil was for diseases of the skin and subcutaneous tissue. Diseases of the digestive system, symptoms, signs and abnormal clinical and laboratory findings not elsewhere classified, spiritual ailments and certain infectious and parasitic diseases received the highest UV for meat, head, tail and foot, respectively. The highest UV of the tongue was for diseases of the circulatory system while the highest UV of the bones was for diseases of the musculoskeletal system and connective tissue.

**Table 2:** **Use Value (UV) of pangolin body parts for each International Classification of Diseases (ICD) category**

	International Classification of Diseases (ICD) categories																	Total
Parts	1	2	3	4	5	6	7	8	9	10	11	12	13	14	15	16	17	UV
Scales	0.063			0.032	0.016	0.016			0.032	0.206	0.143	0.032	0.016	0.079	0.079		0.397	1.111
Oil	0.016						0.016			0.238	0.079			0.079				0.429
Meat	0.016	0.016			0.032		0.048		0.143		0.016		0.063	0.016		0.016		0.365
Head				0.048	0.016		0.016		0.048	0.016	0.016	0.032		0.111	0.016		0.016	0.333
Tail	0.048			0.016	0.032				0.016	0.016	0.032		0.016	0.032	0.016		0.095	0.317
Foot	0.048									0.032				0.016	0.016			0.111
Tongue				0.016			0.048				0.032			0.016				0.111
Bones										0.016	0.063				0.032			0.111
Blood	0.016						0.016		0.016		0.016				0.016		0.032	0.111
Male sex organ	0.032			0.016					0.016			0.016		0.016				0.095
Intestines									0.048					0.016			0.016	0.079
Claws							0.016	0.016		0.016							0.016	0.063
Brain				0.032			0.016		0.016									0.063
Heart							0.016		0.032									0.048
Whole animal	0.016																0.016	0.032
Toes	0.016				0.016													0.032
Thorax											0.016				0.016			0.032
Forefoot	0.016			0.016														0.032
Eyes	0.032																	0.032
Throat			0.016															0.016
Sex organ (both male and female)												0.016						0.016
Liver								0.016										0.016

Codes for ICD categories: 1 = Certain infectious and parasitic diseases, 2 = Diseases of the blood and blood-forming organs and certain disorders involving the immune mechanism, 3 = Endocrine, nutritional and metabolic diseases, 4 = Mental and behavioural disorders, 5 = Diseases of the nervous system, 6 = Diseases of the ear and mastoid process, 7 = Diseases of the circulatory system, 8 = Diseases of the respiratory system, 9 = Diseases of the digestive system, 10 = Diseases of the skin and subcutaneous tissue, 11 = Diseases of the musculoskeletal system and connective tissue, 12 = Diseases of the genitourinary system, 13 = Certain conditions originating in the perinatal period, 14 = Symptoms, signs and abnormal clinical and laboratory findings not elsewhere classified, 15 = Injury, poisoning and certain other consequences of external causes, 16 = Factors influencing health status and contact with health services, 17 = Spiritual ailments.

Table 3 indicates the contribution of each ICD category to the total informant agreement ratio (IAR) of the 22 pangolin body parts. The highest IAR value was recorded for the eyes, which was followed in a descending order by the oil, scale, bones, meat, tail and head. The eyes were only used under certain infectious and parasitic diseases category. The highest IAR value for the oil was linked to diseases of the skin and subcutaneous tissue. The highest IAR value for scales were linked to spiritual ailments and then followed in descending order by diseases of the skin and subcutaneous tissue and diseases of the musculoskeletal system and connective tissue (Table 3). Pangolin bones prescribed for cures relating to diseases of the musculoskeletal system and connective tissue and had the highest IAR value while the highest IAR value of meat was for diseases of the digestive system. The highest IAR value for the tail and head were for cures or treatment relating to spiritual ailments. The claws, whole animal, toes, thorax, fore foot, throat, sex organ (both male and female) and liver recorded a zero IAR value as the number of use reports for each part in each use category is the same. This indicates that there was no agreement between informants on the use of these body parts.

**Table 3:** **Informant Agreement Ratio (IAR) of pangolin body parts for each International Classification of Diseases (ICD) category**

	International Classification of Diseases (ICD) categories																	Total
Parts	1	2	3	4	5	6	7	8	9	10	11	12	13	14	15	16	17	IAR
Scales	0.048			0.024	0.012	0.012			0.024	0.156	0.108	0.024	0.012	0.060	0.060		0.300	0.841
Oil	0.031						0.031			0.470	0.157			0.157				0.846
Meat	0.028	0.028			0.055		0.083		0.249		0.028		0.111	0.028		0.028		0.636
Head				0.079	0.026		0.026		0.079	0.026	0.026	0.052		0.183	0.026		0.026	0.550
Tail	0.079			0.026	0.053				0.026	0.026	0.053		0.026	0.053	0.026		0.158	0.579
Foot	0.214									0.143				0.071	0.071			0.500
Tongue				0.071			0.214				0.143			0.071				0.500
Bones										0.095	0.381				0.190			0.667
Blood	0.024						0.024		0.024		0.024				0.024		0.048	0.167
Male sex organ	0.067	0.000		0.033					0.033			0.033		0.033				0.200
Intestines									0.300					0.100			0.100	0.500
Claws							0.000	0.000		0.000							0.000	0.000
Brain				0.167			0.083		0.083									0.333
Heart							0.167	0.000	0.333									0.500
Whole animal	0.000																0.000	0.000
Toes	0.000				0.000													0.000
Thorax											0.000			0.000				0.000
Forefoot		0.000		0.000														0.000
Eyes	1.000																	1.000
Throat		0.000																0.000
Sex organ (both male and female)												0.000						0.000
Liver								0.000										0.000

Codes for ICD categories: 1 = Certain infectious and parasitic diseases, 2 = Diseases of the blood and blood-forming organs and certain disorders involving the immune mechanism, 3 = Endocrine, nutritional and metabolic diseases, 4 = Mental and behavioural disorders, 5 = Diseases of the nervous system, 6 = Diseases of the ear and mastoid process, 7 = Diseases of the circulatory system, 8 = Diseases of the respiratory system, 9 = Diseases of the digestive system, 10 = Diseases of the skin and subcutaneous tissue, 11 = Diseases of the musculoskeletal system and connective tissue, 12 = Diseases of the genitourinary system, 13 = Certain conditions originating in the perinatal period, 14 = Symptoms, signs and abnormal clinical and laboratory findings not elsewhere classified, 15 = Injury, poisoning and certain other consequences of external causes, 16 = Factors influencing health status and contact with health services, 17 = Spiritual ailments.

There were appreciable differences in body parts ranked according to the three indices (Table 4) where the ranking order varied depending on the index. The UV and UAV indices place scales at the highest rank followed by oil, meat, head and tail for both indices. This was not same for the IAR which ranked the eyes as the most important body part. The throat, sex organ (both male and female) and liver were the lowest in all indices.

**Table 4:** **Evaluation of pangolin body parts using Use Value (UV), Informant Agreement Ratio (IAR) and Use Agreement Value (UAV)**

	Indices			Ranking		
Parts	UV	IAR	UAV	UV	IAR	UAV
Scales	1.111	0.841	0.934	1	3	1
Oil	0.429	0.846	0.363	2	2	2
Meat	0.365	0.636	0.232	3	5	3
Head	0.333	0.550	0.183	4	6	4
Tail	0.317	0.526	0.167	5	7	5
Foot	0.111	0.500	0.056	6	8	7
Tongue	0.111	0.500	0.056	6	8	7
Bones	0.111	0.667	0.074	6	4	6
Blood	0.111	0.167	0.019	6	11	12
Male sex organ	0.095	0.200	0.019	7	10	12
Intestines	0.079	0.500	0.040	8	8	8
Claws	0.063	0.000	0.000	9	12	13
Brain	0.063	0.333	0.021	9	9	11
Heart	0.048	0.500	0.024	10	8	10
Whole animal	0.032	0.000	0.000	11	12	13
Toes	0.032	0.000	0.000	11	12	13
Thorax	0.032	0.000	0.000	11	12	13
Forefoot	0.032	0.000	0.000	11	12	13
Eyes	0.032	1.000	0.032	11	1	9
Throat	0.016	0.000	0.000	12	12	13
Sex organ (both male and female)	0.016	0.000	0.000	12	12	13
Liver	0.016	0.000	0.000	12	12	13

## Discussion

### Use value (UV)

High use values indicate that pangolin body parts are frequently used in traditional healing practices and hold a high level of importance as a source of treatment in patients by local traditional medical practitioners and healers [8, 40]. The high UV for pangolin scales implies that the scales are extremely important to the traditional medical practitioners. Rossato et al. [41] state that a UV of more than 1 indicates that community members or TMPs use this resource for numerous medicinal ailments. The scales were found to be the most medicinally versatile body part, and were applied in 12 out of the 17 (71%) ICD categories. The high use of scales found in this study corroborates with findings by Bräutigam et al. [15] in that a large portion of medical ailments are treated making use of pangolin scales. This was also found to be the case in the Awori people of Nigeria [19]. We found scales to be very important in the treatment of spiritual ailments in addition to the preparation of charms, warding off evil spirits and witchcraft which has also been mentioned in previous studies [15, 17, 19, 24, 25]. Furthermore, the high UV of pangolin scales in treating diseases of the skin and subcutaneous tissue can be attributed the importance attached to pangolin scales by the traditional healers in Sierra Leone. We found a high use of pangolin scales for treating rheumatism which has also been mentioned in previous studies [15, 17, 19, 24] although they did not determine the level of knowledge among their respondents.

The use of pangolin oil in treating diseases has not been previously reported and the UV observed in this study is high and therefore is an indication that it is highly valued by the traditional medical practitioners as a source of medicine in Sierra Leone. Knowledge about pangolin oil as a therapeutic resource was mostly limited to diseases of the skin and subcutaneous tissue that had the highest UV among the five ICD categories. The oil is obtained by placing a pan beneath a pangolin while it is being smoked over a fire. Bräutigam et al. [15] mentions that pangolin meat is less valued in terms of medicinal properties to other body parts, however, our study indicates that the meat was relatively important for the people of Sierra Leone ranking third in terms of its use value. However, the therapeutic properties of the meat are quite specific with knowledge about the meat mostly limited to diseases of the digestive system.

The therapeutic properties of the tail was more limited to spiritual ailments and the relatively low UVs for feet, tongue, bones and blood can be attributed less frequent use by traditional medical practitioners than other body parts for treating ailments. Bones were prescribed primarily for treating musculoskeletal and connective disuse disorders but it has also been reported to treat rheumatism within Nigerian tribal communities [17, 19]. The head was found to be medicinally versatile and is thus involved in treating different diseases under various ICD categories, but was more important to traditional medical practitioners and the local community for the treatment of ailments such as headache, skin rash, fainting (syncope), waist pain, body pain, back pain and body aches. We found that the pangolin head was prescribed within 10 ICD categories, a great deal more than was found in similar studies undertaken in Nigeria by [17, 19]. Furthermore, the thorax and eyes have been reported as having a medicinal use in Nigeria and the tongue, heart and feet have also been used in the Republic of Benin for the treatment of asthma, accelerated heart beating, normal growth and baby vigour [24]. Also, studies in Nigeria indicate the entire animal can be prescribed for invisibility which we did not record in Sierra Leone. It is therefore likely that the prescriptive use of certain pangolin body parts varies between African tribal communities and cultures across the species range.

### Informant agreement ratio (IAR)

The IAR values are a reflection of the extent and distribution of knowledge about the use of pangolins in a community or among a group of people. High consensus is an indication that a particular pangolin part is preferred in the community for treatment of specific ailments [8, 45, 46]. Interestingly, the eyes and not the scales had the highest IAR value and are only used to remedy certain infectious and parasitic diseases. The high IAR for tissue oil and scales can be associated with the high importance and prescriptive use attached to it by the traditional healers. According to Heinrich et al. [47], a high consensus value indicates that the medicinal resource may be an important part of local cultural knowledge. As such, the high IAR for tissue oil to treat diseases of the skin and subcutaneous tissue can be said to be an important remedy within the cultural knowledge of traditional medical practitioners as it scored the highest IAR than all the other categories. Likewise, the high IAR for scales in the treatment of spiritual ailments which was the highest IAR for a single ICD category of any body part can be seen to play a large factor within traditional medical practitioners of Sierra Leone. On the contrary, the low consensus values indicated for claws, whole animal, toes, thorax, fore foot, throat, sex organs and liver may be an indication that these body parts have fallen into disuse because of cultural adaptation or believed to be ineffective for treating conditions reported or may simply be of low cultural importance in traditional medicine. Even though a medical resource may be mentioned in traditional pharmacopoeia, this does not necessarily mean that it is perceived as an effective treatment of a condition or alleviating symptoms [44]. This may be the case with regards body parts with low use values and informant agreement ratios and may be an indication that those parts are not effective in treating conditions or alleviating symptom although they occur in within this traditional pharmacopoeia.

One disease that was surprisingly not mentioned but that occurs in the study area and affects a large number of locals but not treated with pangolin parts is malaria. The selection and use of a resource for traditional medicinal purposes are usually based on culturally perceived effectiveness [48]. The lack of effectiveness of pangolin body parts for the treatment of malaria may have accounted for its noticeable absence in the list of diseases mentioned by traditional medical practitioners.

### Comparing different indices

Both the UV and IAR varied for different ICD categories. Thomas et al. [44] attributes this variation to the idiosyncratic knowledge of most traditional healers resulting in low levels of consensus for use categories among practitioners. According to Thomas et al. [44], the distribution of knowledge about traditional remedies follows a pattern whereby few remedies are known to almost everyone while most knowledge is idiosyncratic. All the TMPs interviewed mentioned that they obtained their knowledge verbally from a family member (mother, father, uncle and aunt). Thus their medicinal knowledge reflects knowledge acquired and accumulated over time and that it is likely to be family-specific or idiosyncratic in nature.

Based on the ranking of the indices, the scales, oil, meat, head, tail, foot, tongue and bones were the most widespread body parts used for medicinal purposes although their ranking varied depending on the chosen index. The combination of the number of use reports and the level of consensus between participants seems to provide a valid and easily derived estimate of cultural significant [44] and, it can therefore be deduced that the culturally important pangolin body parts found in this study are those with high use agreement values. The scales had the largest average number of use reports and were used to treat more ICD categories than any other body part. It can therefore be argued, based on the index of use agreement value, that the cultural importance of pangolin scales in traditional pharmacopoeia is very high within Sierra Leone. The other culturally significant pangolin body parts used for medicinal purposes in descending order are oil, meat, head, tail, foot, tongue and bones.

### Conservation implications

Undoubtedly, the high dependency on traditional medicine as well as high level of consensus among healers on the choice of body parts to be used in treating various ailments raises concern about the level of harvesting of pangolins for medicinal purposes in Sierra Leone. All the significant body parts based on the UAV are used to cure culturally bound syndromes as well as diseases that have been identified as prevalent in Sierra Leone. Culture-bound syndromes or folk illness usually have no substituted or alternative remedies with healers relying on what they are culturally familiar with. For instance, pangolin body parts used in the form of amulets and charms in the treatment of spiritual ailments cannot be substituted with another animal or provided for clinically. The belief systems may not provide an option for another alternative medicine, making sole reliance on pangolin body parts inevitable. According to Costa-Neto [49], cultural medical systems are often organised within cultural systems and the use of animals or parts thereof within these medical systems should be understood from a cultural perspective. Often, the effectiveness of traditional medicinal resources is not simply a consequence of their pharmacology but also stems from a cultural and traditional background and belief system of a tribe or community [50]. This study has indicated that particular pangolin body parts, such as scales, have an important cultural medicinal value and are prescribed frequently within Sierra Leon. The harvest of pangolins is further increased by the high use values for treatment of diseases of the digestive system, diseases of the skin and subcutaneous tissue and diseases of the musculoskeletal system and connective tissue that have been identified as very prevalent in Sierra Leone by the WHO [51, 52]. Furthermore, none of the body parts used to treat conditions under these ICD categories can be obtained without killing the animal which further raises concerns about the impact of harvesting pressure on these animals. Also, there are no cultural taboos associated with the traditional medicinal use as well as harvesting of pangolins at anytime of the year among traditional medical practitioners interviewed in Sierra Leone. Pangolins can subsequently be regarded as *highly sought after* for traditional medicinal practices and actively pursued or poached in this country. As such, an assessment of the cultural dimension of pangolin use in traditional medicine is critical for conservation efforts for the species. The actual levels of harvest or trade is not documented neither is the turn-over rates within bush meat markets or medical practitioner use in this country. Furthermore, the extent of international trade, particularly towards Asia, is on the rise. Recently, two shipments of pangolin scales originating from East and West Africa, respectively, with a combined mass of 3.3 tonnes were intercepted in Hong Kong harbour by customs officials during May and June 2014 [53].

All three species of African pangolin occurring within Sierra Leone; black-bellied pangolin (*Phataginus tetradactyla*), white-bellied pangolin (*Phataginus tricuspis*) and the giant ground pangolin (*Smutsia gigantea*) are listed on CITES Appendix II and can only be traded following a Non-Detriment Finding study that has been completed and logged. To the best of our knowledge this has never been done for any of the African species. Furthermore, all three species have been up-listed as *Vulnerable* on the recently revised IUCN Red List [34]. All three pangolin species are protected under the Wildlife Conservation Act of 1972 in this country and offenders of a first conviction may be fined or sentenced with a prison term not exceeding six months, or both may apply. For a second and subsequent conviction a fine or a prison term not exceeding 12 months, or both, may be imposed. However, this is rarely imposed and in addition to the poor enforcement of conservation laws, as in most developing countries, it does not contribute to the conservation of pangolins.

Currently, there is little data that assesses the status and use of African species of pangolins when compared to Asian species. Alves et al. [54] have identified the need to increase our understanding of the biology and ecology of species commonly used as remedies to better assess the impact of harvesting them for medicinal or other purposes on their wild populations. This is critical for pangolins since all the species used by traditional medical practitioners in this study and studies elsewhere [17, 19] were harvested from the wild with no known record of captive breeding or domestication. Based on their habitat and prey requirements, reproductive rates, population distribution, degree of habitat alteration, levels of hunting pressure and medicinal value, all pangolin species are susceptible to extinction [22, 34]. High demand for pangolins from the wild may lead to a strong possibility of overexploitation through medicinal use, which requires an urgent ecological evaluation of the population in the wild. The impact of animal-based medicinal practices on wild populations therefore needs to be carefully assessed considering that preparation of most animal-based remedies requires killing of the animal.

Conservation efforts should therefore be aimed at educating as traditional medical practitioners about the implications of their medical practices on the wild population. Traditional medical practitioners must be made aware that a decrease in medicinal wildlife not only affects biodiversity but will jeopardise their socioeconomic status since the raw materials they rely on to treat the patients will no longer be available. It is therefore imperative for them to ensure the continuous survival of these animals through sustainable utilization of the wild population.

## Conclusion

Traditional medicine represents an alternative source of healthcare for most people in Sierra Leone and knowledge about the medicinal properties of pangolins is very customary among the local people as well as traditional medical practitioners. This was evident in the high cultural and therapeutic importance associated with pangolin body parts. The folk culture of the people is the key factor in maintaining and driving the use of pangolins for therapeutic purposes. The lack of enforcement of wildlife conservation laws is negatively affecting the sustainable harvesting of pangolins as it is currently very likely an over exploitation of a threatened and vulnerable species. Collaboration between conservation agencies and traditional medical practitioners through the involvement of the local community is necessary to prevent a population crash of three African pangolin species which will, in turn, have an impact on the cultural use of the species. Conservation efforts should therefore be aimed at increasing awareness among traditional medical practitioners and soliciting their support to ensure the success of conservation programmes and sustainable pangolin use. Further studies with regards the use of pangolins as therapeutic resources is necessary in other African countries to increase our understanding of the requirement for the species as a source of traditional medicine in addition to documenting the potential impact on the species with regards to local harvest levels.
